# Involvement of Cold Inducible RNA-Binding Protein in Severe Hypoxia-Induced Growth Arrest of Neural Stem Cells In Vitro

**DOI:** 10.1007/s12035-016-9761-1

**Published:** 2016-03-01

**Authors:** Qian Zhang, Ya-Zhou Wang, Wenbin Zhang, Xiaoming Chen, Jiye Wang, Jingyuan Chen, Wenjing Luo

**Affiliations:** 1 Department of Occupational and Environmental Health, the Ministry of Education Key Lab of Hazard Assessment and Control in Special Operational Environment, School of Public Health, Fourth Military Medical University, 169 Chang Le Xi Road, Xi’an, Shaanxi 710032 China; 2 Department of Neurobiology and Collaborative Innovation Center for Brain Science, School of Basic Medicine, Fourth Military Medical University, 169 Chang Le Xi Road, Xi’an, Shaanxi 710032 China

**Keywords:** Severe hypoxia, Neural stem cells, CIRBP, Proliferation

## Abstract

Neonatal hypoxia is the leading cause of brain damage with birth complications. Many studies have reported proliferation-promoting effect of mild hypoxia on neural stem cells (NSCs). However, how severe hypoxia influences the behavior of NSCs has been poorly explored. In the present study, we investigated the effects of 5, 3, and 1 % oxygen exposure on NSCs in vitro. MTT, neurosphere assay, and 5-ethynyl-2′-deoxyuridine (EdU) incorporation revealed a quick growth arrest of C17.2 cells and primary NSCs induced by 1 % oxygen exposure. Cell cycle analysis showed that this hypoxia exposure caused a significant increase of cells in G0/G1 phase and decrease of cells in S phase that is associated with decrease of Cyclin D1. Interestingly, the expression of cold inducible RNA-binding protein (CIRBP), a cold responsive gene reacting to multiple cellular stresses, was decreased in parallel with the 1 % oxygen-induced proliferation inhibition. Forced expression of CIRBP under hypoxia could restore the proliferation of NSCs, as showed by EdU incorporation and cell cycle analysis. Furthermore, the expression of Cyclin D1 under hypoxia was also restored by CIRBP overexpression. Taken together, these data suggested a growth-suppressing effect of severe hypoxia on NSCs and, for the first time, revealed a novel role of CIRBP in hypoxia-induced cell cycle arrest, suggesting that modulating CIRBP may be utilized for preventing hypoxia-induced neonatal brain injury.

## Introduction

Neonatal hypoxia usually leads to permanent neurological handicap, practically because the immature brain is at a critical state of dynamic development. Extensive studies have been conducted on the apoptosis-inducing effects of hypoxia on neurons and oligodendrocytes [1–4]. Neural stem cells (NSCs), the major cell type that bears the capacity of regenerating lost nerve cells, are mainly reserved in the subventricular zone (SVZ). In rodents, the region of SVZ is at its maximum size at neonatal stage [5]. Therefore, it is of importance to explore the effects of neonatal hypoxia on the behavior of NSCs. In comparison with the oxygen tension in blood which is 14 %, brain tissue is exposed to hypoxia, with oxygen tension varies from 0.55 % in the midbrain to 8 % in regions near the pia [6, 7]. Therefore, it has been proposed that hypoxia may be beneficial for NSCs, as some studies have showed that mild hypoxia (2.5–5 %), mimicking the in vivo oxygen level of embryonic brain, increases the proliferation of NSCs [8–10]. However, the long-term neurogenesis deficiency observed after neonatal hypoxia stress strongly suggests a probably detrimental effect of severe hypoxia (<1 %), which occurs under pathological conditions such as ischemia and tumor growth [6, 7, 11–14], on the proliferation of NSCs which remains poorly investigated.

Cold inducible RNA-binding protein (CIRBP) is initially identified as a member of cold shock proteins and can be induced by multiple stressful conditions such as moderate hypothermia, hypoxia, and ultraviolet irradiation [3, 15–20]. Later studies revealed its participation as RNA chaperones in circadian rhythm regulation, neural development, tumorigenesis, and inflammation [21–24]. Of note, recent studies have demonstrated an involvement of CIRBP in the proliferation of tumor cells and germ cells [25–30]. Under low temperature, CIRBP is reportedly activated and contributed to the preservation of the stemness of NSCs [31]. How CIRBP is regulated and involved in the response of neural stem cells to severe hypoxia remains largely unclear.

In the present study, we explored the effects of 1 % O_2_, a condition which has been adopted to mimic the ischemic attachment in vivo [12, 19, 32], on the proliferation of NSCs and expression of CIRBP in NSCs and further examined the role of CIRBP in neural stem cells under severe hypoxia.

## Materials and Methods

### Cell Culture

Mouse NSC line C17.2 [33] was cultured in Dulbecco’s modified Eagle’s medium (DMEM) (Invitrogen) containing 2 mM l-glutamine (Invitrogen), 10 % fetal bovine serum (FBS) (Sijiqing Biotech, China), 5 % horse serum (Gibco), 100 units/mL of penicillin, and 100 μg/mL of streptomycin. The cultures were maintained in a standard humidified incubator in 5 % CO_2_ at 37 °C, with fresh medium replaced every 2 days, and split 1:4 when the cells reached 90 % confluence.

Primary neural stem cells were isolated from newborn SD rat cerebral cortex and cultured in uncoated 25-mL flasks in DMEM/F-12 medium (Invitrogen) containing N2 and B27 supplements (Invitrogen) plus basic fibroblast growth factor (Promega, 20 ng/mL) and epidermal growth factor (Promega, 20 ng/mL). After 5–7 days culture in vitro (DIV), the primary neurospheres were collected and dissociated with 0.05 % trypsin plus 200 mM EDTA for 10 min at 37 °C and mechanically triturated with fire-polished glass pipettes. The single cells were resuspended at a density of 50,000 cells per mL of serum-free medium and cultured for 3–5 days. The number and diameters of neurospheres were assessed as described [34].

### Hypoxia Exposure

Hypoxia environment was made by placing cells in a humidified microaerophilic incubation system (DWS HypOxystation) with a calibrated gas containing 1 % O_2_ or 3 % O_2_ at 37 °C (CO_2_ was adjusted at 5 % in both conditions). The cells were left in the incubator at 37 °C for different durations. The control cultures were incubated in normoxic conditions all the time for the same durations.

### MTT Assay

C17.2 NSCs were plated in 96-well plates at 5000 cells per well in growth media cultured in DWS HypOxystation incubator perfused with a calibrated gas mixture of 1 % O_2_ or 3 % O_2_ with 5 % CO_2_ at 37 °C or in a normal incubator with 21 % O_2_ and 5 % CO_2_ at 37 °C for 0, 12, 24, 36, and 48 h, respectively. Twenty microliters of MTT stock solution (5 mg/mL) was added to the medium for 4 h at 37 °C. Then the supernatant was removed and replaced with 150 μL of dimethyl sulfoxide (DMSO) for 10 min at 37 °C until crystals were dissolved. The plates were shaken vigorously for 10 min to ensure complete dissolvent. MTT quantification was measured at 490 nm with a microplate reader.

### EdU Incorporation Assay

The EdU incorporation assay was performed with a Cell-Light EdU kit (Ribobio Co., Ltd., Guangzhou, China) according to the manufacturer’s instructions. Briefly, C17.2 NSCs were cultured in six-well plates coated with poly-d-lysine at a cell density of 1.5 × 10^5^ cells per well, and the cells were then labeled with 50 μM EdU (1:1000) and incubated for an additional 2 h before the cells were fixed with 4 % formaldehyde for 15 min at room temperature and treated with 0.5 % Triton X-100 for 20 min at room temperature for permeabilization. After washing with PBS for three times, each well of cells was reacted with 100 μL of 1× ApolloIV reaction cocktail for 30 min. Subsequently, the nucleus was counterstained with Hoechst 33342 and the cells were observed using a fluorescence microscope (IX70, Olympus).

### Plasmid Construction and Transfection

Human CIRBP complementary DNA (cDNA) clone (NC_000019) in pEGFP-N2 vector was a gift from Dr. Wenbin Zhang in the lab. The control transfection was performed by pEGFP-N2 vector without CIRBP. Overexpression of CIRBP by these cells was verified by Western blotting using anti-CIRBP antibody as described below.

Transfection of C17.2 neural stem cells with CIRBP cDNA was performed by using Lipofectamine 2000 transfection reagent (Invitrogen, USA) according to the manufacturer’s procedure. In brief, cells were plated in six-well plates (Nunc) at a cell density of 10,000 cells per well and were allowed to grow overnight to achieve 80 % confluency. Transfection complexes, consisting of 2.5 μg pEGFP-N2 vector plasmid DNA or pEGFP-N2-CIRBP plasmid DNA and 6 μL Lipofectamine reagent, were added to the wells in Opti-MEM® Medium (Invitrogen, USA). Cells were analyzed 24 h after lipofection for transfection efficiency and viability.

Transfection of primary neural stem cells with CIRBP cDNA was performed by electroporation. In brief, the primary neurospheres were collected when diameters ranged between 80 and 120 μm, and dissociated with 0.05 % trypsin and 200 mM EDTA for 10 min at 37 °C, and mechanically triturated with fire-polished glass pipettes. The single cells were resuspended at a density of 1 × 10^7^ cells per mL of serum-free Opti-MEM® medium. Mixture of the single cell suspension with plasmid DNA makes its final concentration reached 1 × 10^6^ cells and 10 μg plasmid DNA (1 μg/μL) in 100 μL solutions. Two pulses of 125 V, 7.5 ms each at 50 ms intervals, were delivered through electroporation cuvettes (2 mm gap) with a NEPA21 electroporator. After electroporation, the mixture in the cuvette was supplemented with 2 mL serum-free neural stem cell media and transferred gently into a prepared uncoated six-well plate. Cells were analyzed 48 h after electroporation for transfection efficiency and viability.

### Real-time Reverse Transcription-PCR

Cells were harvested for total RNA isolation using Trizol reagent (Invitrogen) according to manufacturer’s instructions. After reverse transcription, real-time PCR was performed using ABI7500 system. The reaction was made in 10 μL of SYBR Green I (Takara), 0.5 μM of each 5′ and 3′ primer, 2 μL cDNA, and H_2_O to a final volume of 20 μL. Samples were amplified for 40 cycles with a denaturation at 95 °C for 5 s, annealing, and extension at 57.5 °C for 34 s. SYBR green fluorescence was measured to determine the amount of double-stranded DNA. To discriminate specific from nonspecific cDNA products, a melting curve was obtained at the end of each run. Relative messenger RNA (mRNA) levels of target genes were normalized to GAPDH and compared with the control using the 2^−ddCt^ methods. The primer sequences used in this study were listed below.

For rat CIRBP,

5′-TTACTGTTTACCATGAGCCATG-3′ (forward) and 5′-CACACAACCCGACAATTTAG-3′ (reverse).

For rat glyceraldehyde-3-phosphate dehydrogenase (GAPDH),

5′-TACCCACGGCAAGTTCAACG-3′ (forward) and 5′-CACCAGCATCACCCCATTTG-3′ (reverse).

For mouse CIRBP,

5′-TCCAGAGACTACTATGCCAG-3′ (forward) and 5′-GAACGGAAAGGACTACAAAA-3′ (reverse).

For mouse glyceraldehyde-3-phosphate dehydrogenase (GAPDH),

5′-AATGGTGAAGGTCGGTGTGA-3′ (forward) and 5′-GCTCCTGGAAGATGGTGATG-3′ (reverse).

For mouse Cyclin D1,

5′-GGATGCTGGAGGTCTGTGAG-3′ (forward) and 5′-CGGCAGTCAAGGGAATGGTC-3′ (reverse).

### Cell Cycle Analysis

For analysis of cell cycle, cells with different treatments were trypsinized, washed twice in PBS, and fixed overnight at −20 °C in 300 μL PBS and 700 μL ethanol. The fixed cells were spun down gently in 200 μL extraction buffer (0.1 % Triton X-100, 45 mM Na_2_HPO_4_ and 2.5 mM sodium citrate) at 37 °C for 20 min and then stained with propidium iodide (BD Biosciences, San Jose, CA, USA) (50 μg/mL) containing 50 μg/mL RNase A for 30 min at 37 °C in the dark, and subsequently analyzed by FACS. The experiment was repeated for at least three times, and the data were analyzed using CellQuestk and ModFitk software.

### Western Blotting Analysis

Cells were washed twice with ice-cold PBS and lysed with buffer containing Tris-HCl (50 mM, pH = 7.4), NP-40 (1 %), Na-deoxycholate (0.25 %), NaCl (150 mM), EDTA (1 mM), PMSF (1 mM), Na_3_VO_4_ (1 mM), and NaF (1 mM) for total extract. Protein concentration was determined by the BCA protein assay (Pierce Chemical Co.). Equal amounts of cell lysates were separated by 10 % SDS-polyacrylamide gel electrophoresis and electro-transferred onto nitrocellulose membranes. Membranes were then incubated in blocking solution (5 % nonfat milk in 20 mM Tris-HCl, 150 mM NaCl, 0.1 % Tween-20) (TBS-T), followed by incubation with the indicated antibodies at 4 °C overnight. The membranes were then washed in TBS-T and incubated with HRP-conjugated secondary antibodies for 1 h at room temperature. Enhanced chemiluminescence (ECL) Western blotting substrate (Pierce) was used to detect the immunoreactive signals with an ECL-based FluorChem FC2 image system (Alpha Innotech). Rabbit anti-CIRBP was purchased from ProteinTech, and rabbit anti-Cyclin D1 was from Santa Cruz Biotechnology. All Western blotting analyses were performed in triplicates. FluorChem FC2 software was used to analyze the gray values of the bands in each group.

### Statistics

Data were presented as the mean ± S.E. Statistical analysis of the data for multiple comparisons was performed by analysis of variance. *T* test and ANOVA were adopted for comparison. A value of *p* < 0.05 was considered statistically significant.

## Results

### Severe Hypoxia Inhibits Proliferation of NSCs

**Fig. 1:**
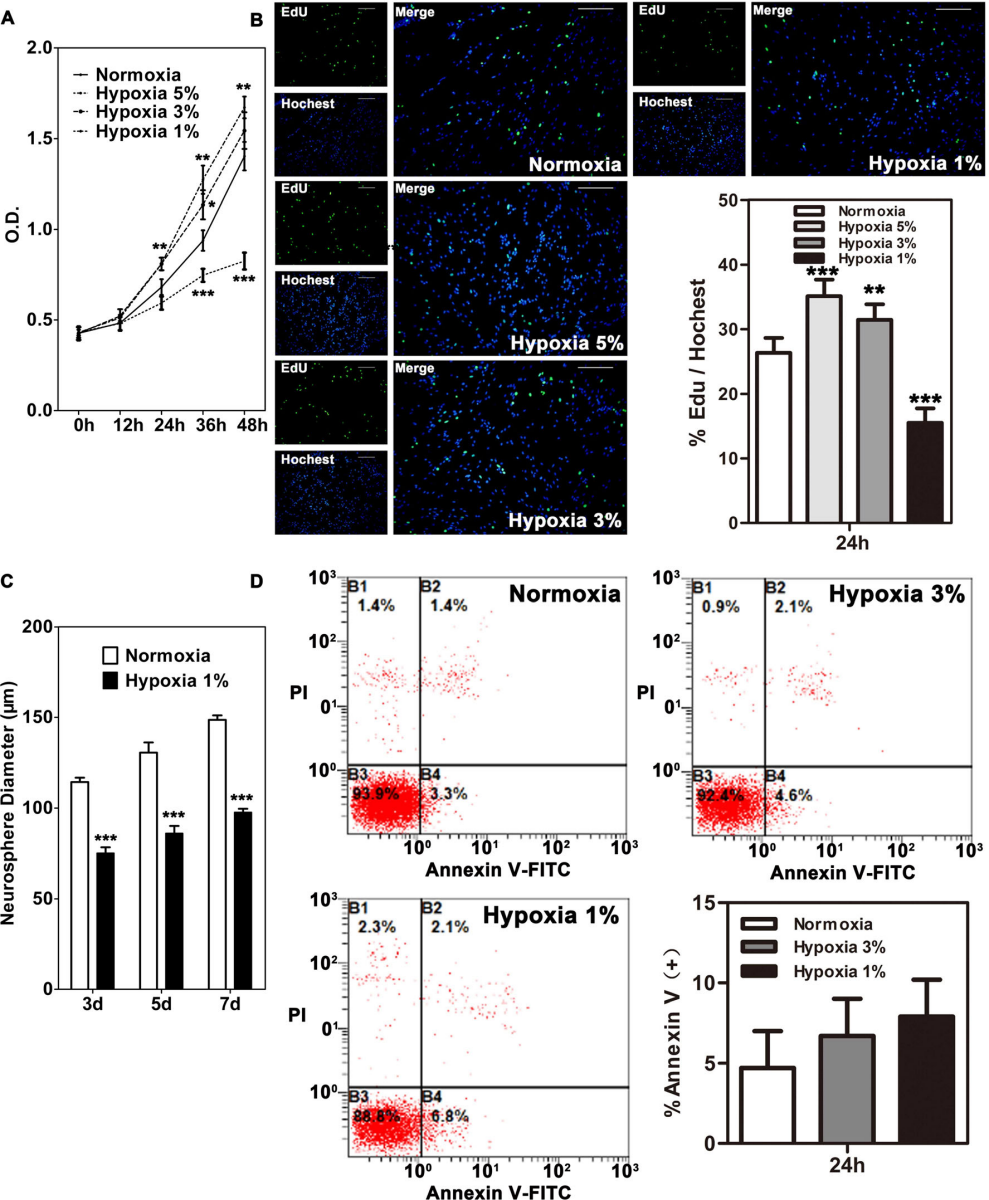
Effects of hypoxia on the proliferation of NSCs. **a** C17.2-NSC cells were cultured under normoxia or hypoxia for 0, 12, 24, 36, and 48 h. Cell proliferation was assessed using MTT assay. Notice the increase of O.D. values by 5 % O_2_ and decrease of O.D. values by 1 % O_2_. *** depicts significant decrease of O.D. values after exposure to hypoxia (*p* < 0.001). **b** EdU immunostaining of C17.2 NSCs cultured under normoxic or hypoxic conditions for 24 h and quantification of EdU+ cells. ** indicates a significant decrease of EdU+ cells in NSCs cultured with 1 % O_2_ (*p* < 0.001). *Bars* = 100 μm. **c** Effect of 1 % O_2_ on the formation of neurospheres. Primary neural stem/progenitor cells were cultured in normoxia or 1 % O_2_ for 7 days. The diameters of neuospheres were measured on days 3, 5, and 7. *** depicts smaller neurospheres in cells cultured with 1 % O_2_ than those in cells in normoxia (*p* < 0.001). **d** Effect of 1 % O_2_ or 3 % O_2_ on the apoptosis of NSCs after 24 h exposure to hypoxia

We first evaluated the effects of 5, 3, and 1 % O_2_ on the growth of C17.2 NSCs. MTT assay was adopted to examine the cell viability at various time points (0, 12, 24, 36, 48 h) after exposure to hypoxia. From 36 h, the O.D. values in cells treated with 5 % were significantly higher than those in cells cultured in normal condition (Fig. 1a). Interestingly, the O.D. values in 3 % O_2_-treated cells showed a slight increase at 36 h but no significant change at 48 h exposure to hypoxia (Fig. 1a). In contrast, the O.D. values of cells in 1 % O_2_ became significantly lower than normal control from 36 h exposure (Fig. 1a). To assess the effects of hypoxia on the proliferation of C17.2 NSCs, we performed 5-ethynyl-2′-deoxyuridine (EdU) incorporation assay. At 24 h after hypoxia, percentages of EbU-labeled cells in 5 and 3 % O_2_ were significantly increased, but were significantly decreased in cells cultured by 1 % O_2_ (Fig. 1b). These results indicate that 1 % O_2_ may inhibit the growth of NSCs. To further confirm the growth-inhibiting effect of 1 % O_2_ on NSCs, we cultured primary neural stem/progenitor cells in two different oxygen concentrations (21 % O_2_ normoxia group vs 1 % O_2_ hypoxia group) and examined the size of neurospheres. The diameters of neurospheres on days 3, 5, and 7 days in vitro (DIV) were measured and compared. Primary neural stem/progenitor cells gave rise to smaller neurosphere in 1 % O_2_ than those cultured in normoxia with mean neurosphere diameters of 75.13 ± 31.21 μm vs 114.37 ± 39.55 μm on day 3, 86.11 ± 27.67 μm vs 130.67 ± 46.46 μm on day 5, and 97.68 ± 26.00 μm vs 148.60 ± 45.50 μm on day 7 (Fig. 1c).

To examine whether 1 % O_2_ affected the survival of NSCs, we analyzed the expression of annexin-V, a marker of apoptosis. No significant difference of annexin-V expression was detected among cells in normal condition, 1 % O_2_, and 3 % O_2_ after 24 h exposure when EdU incorporation has been reduced in 1 % O_2_ (Fig. 1d). Therefore, it seems that 1 % O_2_ may induce growth arrest of NSCs without affecting cell survival.

### Cell Cycle Arrest of C17.2 NSCs After Severe Hypoxic Stimulation

**Fig. 2:**
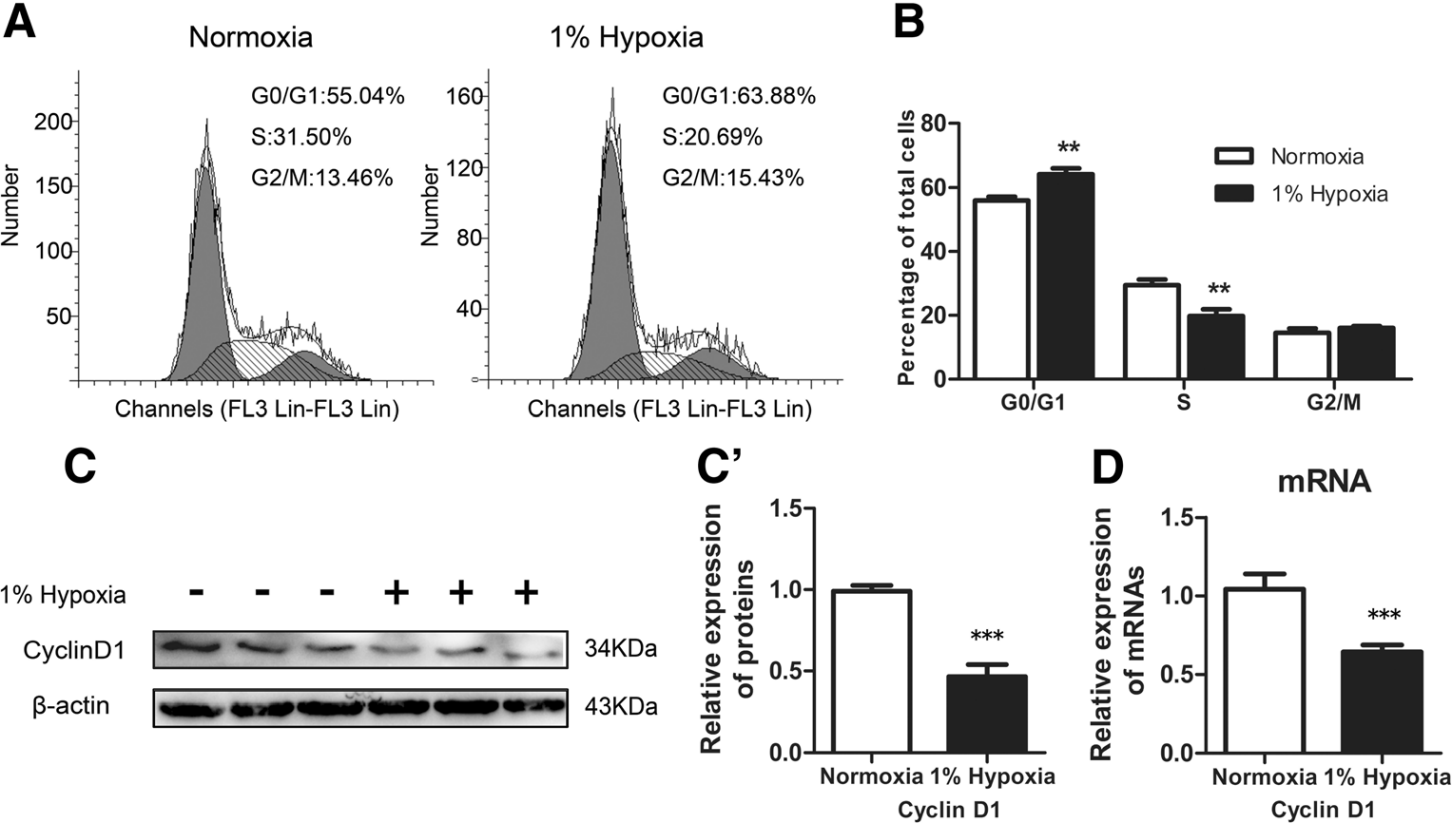
Effect of 1 % O_2_ on the cell cycle of C17.2 NSCs. **a** A representative plot of cell cycle from one of three independent experiments. The first *gray* area (*close to Y axis*), the *hatched* area, and the second *gray* area (*distant to Y axis*) represent the number of cells in G1/G0, S phase, and G2 phase, respectively. **b** Percentages of cells in G1/G0, S, and G2/M phase in control and hypoxia-exposed groups. ** indicates significant difference between the control and hypoxia-exposed groups (*p* < 0.01). **c** Western blotting analysis of Cyclin D1 in NSCs cultured in normoxia or 1 % O_2_ for 24 h (*left*). Samples in each group were from three independent experiments. The relative expression levels of Cyclin D1 protein were quantified (*right*). β-actin served as a protein loading control. **d** Real-time RT-PCR analysis of Cyclin D1 mRNA in NSCs cultured in normoxia or 1 % O_2_ for 24 h. GAPDH served as an internal control. *** depicts the significant decrease of Cyclin D1 at both protein (**c**) and mRNA (**d**) levels after severe hypoxia exposure (*p* < 0.001)

We then focused on the effects of 1 % O_2_ on the proliferation of NSCs. Cell cycle analysis was performed in both control and 1 % O_2_-treated C17.2 NSCs. In the control group, approximately 55.04 % of cells stayed at in G1 phase, 31.50 % at S phase, and 13.46 % at G2/M phase. In the hypoxia exposure group, approximately 63.88 % of cells were in G1 phase, 20.69 % in S phase, and 15.43 % in G2/M phase (Fig. 2a). Statistical analysis showed a significant increase of cells in G0/G1 and decrease of cells in S phase by 1 % O_2_ (Fig. 2b).

To elucidate the molecular mechanisms underlying hypoxia-induced cell cycle arrest, we examined the expression levels of Cyclin D1 that plays a crucial role in controlling cell cycle progression from G0–G1 to S phase and acts as a sensor for cell cycle machinery in response to stress, cytokine, and growth factor stimulation. As shown in Fig. 2c, the expression of Cyclin D1 protein was significantly decreased in cells exposed to hypoxia for 24 h (Fig. 2c). Reduction of Cyclin D1 was further confirmed by real-time RT-PCR analysis that showed decreased levels of Cyclin D1 mRNA in hypoxia-exposed cells (Fig. 2d). Taken together, these data suggest that 1 % O_2_ might induce the cell cycle arrest of NSCs.

### Severe Hypoxia Exposure Decreases the Expression of CIRBP in NSCs

**Fig. 3:**
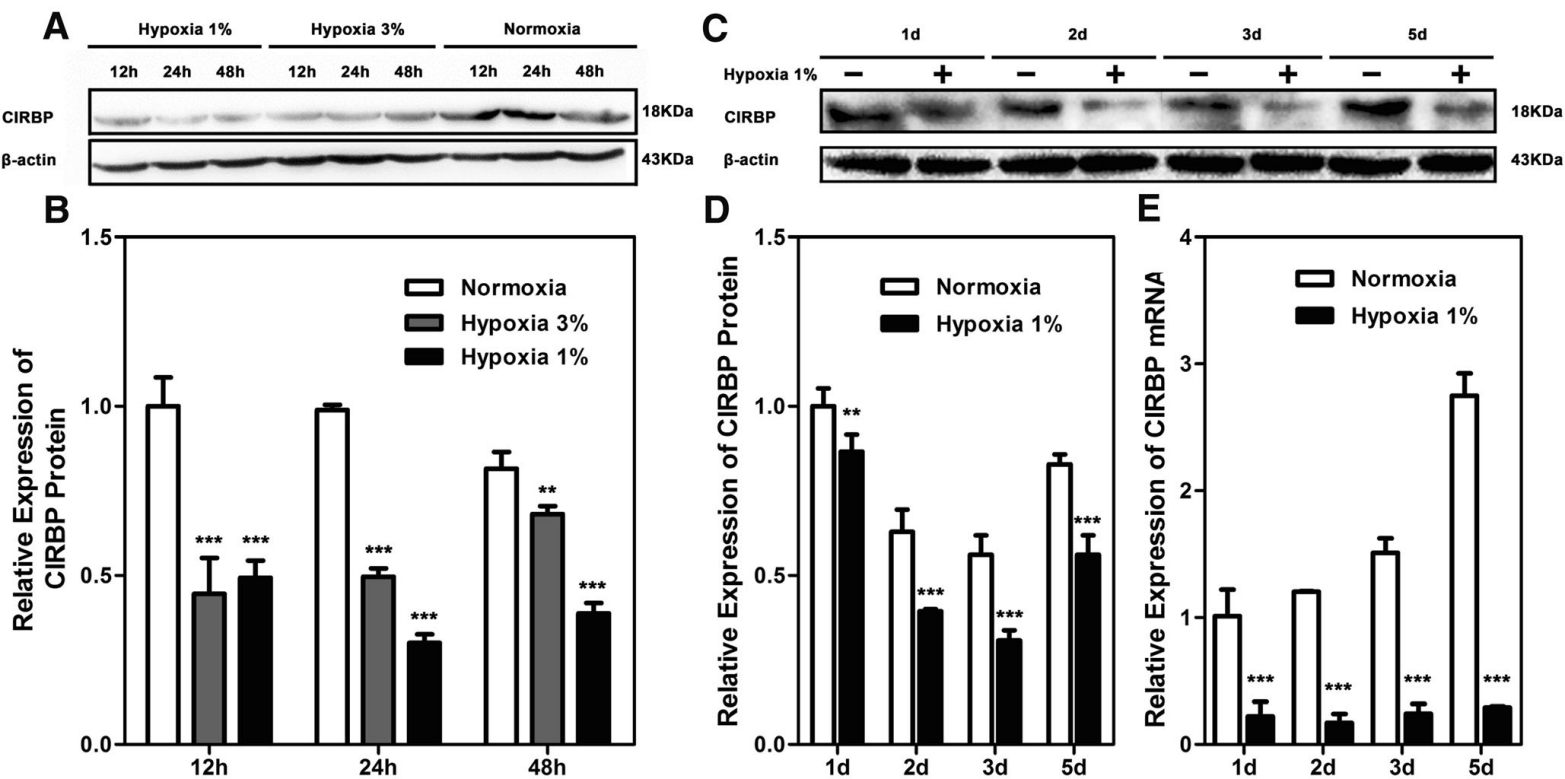
Effect of hypoxia on the expression of CIRBP in NSCs. **a**, **b** Expression of CIRBP in C17.2 NSCs after exposure to 1 or 3 % O_2_. Notice the suppression of CIRBP in 1 % O_2_ and the transient suppression of CIRBP in 3 % O_2_. **a**–**d** Expression of CIRBP in primary NSCs at different time points after exposure to 1 % O_2_. **e** Real-time RT-PCR of CIRBP in primary NSCs at different time points after exposure to 1 % O_2_. Notice the decrease of CIRBP both in protein and in mRNA after exposure to 1 % O_2_. *** depicts the significant decrease of CIRBP levels after hypoxia treatment (*p* < 0.001)

To explore the possible mechanism for the severe hypoxia-induced growth arrest of NSCs, we examined the expression of CIRBP, a stress response protein that has been thought to be involved in regulating the proliferation of tumor cells [25, 27], in C17.2 NSCs under the conditions of hypoxia. Western blotting showed a significant decrease of CIRBP at 24 h in 3 % O_2_ which recovered to approximately 80 % of the normal expression level at 48 h (Fig. 3a, b).

The effect of 1 % O_2_ on the expression of CIRBP was further investigated using primary neural stem cells that were cultured under normoxia or 1 % O_2_ for 1, 2, 3, and 5 days. The levels of CIRBP protein in hypoxia-exposed cells at all time points were significantly lower than those in normoxia groups (Fig. 3c, d). In addition, a significantly lower level of CIRBP mRNA was also detected in 1 % hypoxia-treated primary neural cells from 24 h to 5 days exposure (Fig. 3e). These data demonstrated that severe hypoxia exposure decreased the expression of CIRBP in both C17.2 NSCs and primary NSCs, indicating CIRBP may be involved in the growth inhibitory effects of hypoxia on NSCs.

### Overexpression of CIRBP Rescues the Growth Arrest of NSCs Caused by Severe Hypoxia

**Fig. 4:**
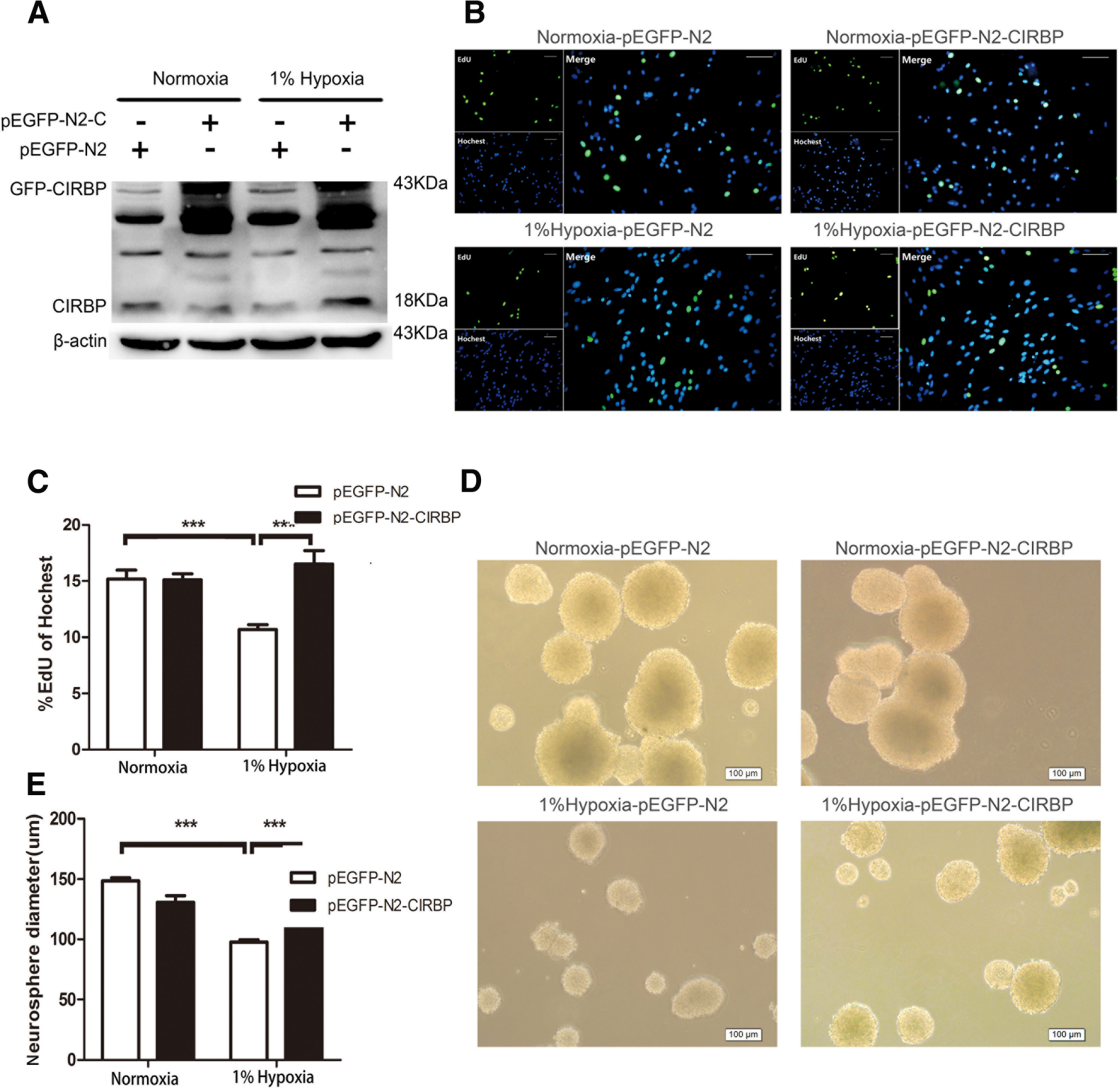
Effect of CIRBP overexpression on the hypoxia-induced growth suppression of NSCs. **a** Western blotting analysis of CIRBP protein levels in C17.2 NSCs transfected with mock vector (pEGFP-N2) or pEGFP-N2-CIRBP (pEGFP-N2-C) that were cultured in normoxia or 1 % O_2_ for 24 h. **b**, **c** EdU immunostaining of transfected C17.2 NSCs cultured in normoxia or 1 % O_2_ for 24 h and quantification of EdU+ cells. *Bars* = 100 μm. **d**, **e** Size of neurospheres formed from primary NSCs transfected with mock vector or pEGFP-N2-CIRBP cultured in normal condition or under 1 % O_2_. *** indicates a significant decrease of Edu+ cells (**c**) and neurosphere size (**e**) in mock vector-transfected cells cultured with 1 % O_2_, but a significant increase of Edu+ cells (**c**) and neurosphere size (**e**) in pEGFP-N2-CIRBP-transfected cells as compared to mock vector-transfected cells under hypoxia (*p* < 0.001)

To investigate the potential link between the reduction of CIRBP and hypoxia-induced NSC growth arrest, we transfected C17.2 NSCs with p-EGFP-N2-CIRBP plasmid and examined the cell proliferation by EdU incorporation assay after exposure to 1 % O_2_ for 24 h. Overexpression of CIRBP in transfected cells was confirmed by Western blotting (Fig. 4a). Under normoxic culture condition, there were no significant differences of the percentages of EdU-positive cells between mock vector- and p-EGFP-N2-CIRBP-transfected cell groups. Under 1 % O_2_ hypoxia, overexpression of CIRBP increased the percentages of EdU-positive cells from 10.68 to 16.52 % (Fig. 4b, c). These results suggest that the overexpression of CIRBP may restore the proliferation of C17.2 NSCs under 1 % O_2_ hypoxia.

We next transfected primary cultured NSCs with p-EGFP-N2-CIRBP plasmids by electroporation, and then exposed the cells to 1 % O_2_ for 5 days and examined the changes of neurosphere diameters. The transfection efficiency was about 70 % (data not shown). Under normoxia, the mean neurosphere diameters were 144.32 ± 43.29 μm in p-EGFP-N2-CIRBP-transfected cells and 128.67 ± 38.36 μm in the mock vector-transfected cells. Under hypoxia, the mean neurosphere diameters were 112.05 ± 37.82 μm in the p-EGFP-N2-CIRBP-transfected cells and 95.45 ± 29.37 μm in the mock vector-transfected cells. CIRBP overexpression significantly increased the size of neurospheres under 1 % O_2_ (Fig. 4d, e). Together, these data suggest that the overexpression of CIRBP may rescue the severe hypoxia-induced growth arrest of NSCs.

### Effects of CIRBP Overexpression on the Cell Cycle Arrest of NSCs Induced by Severe Hypoxia

**Fig. 5:**
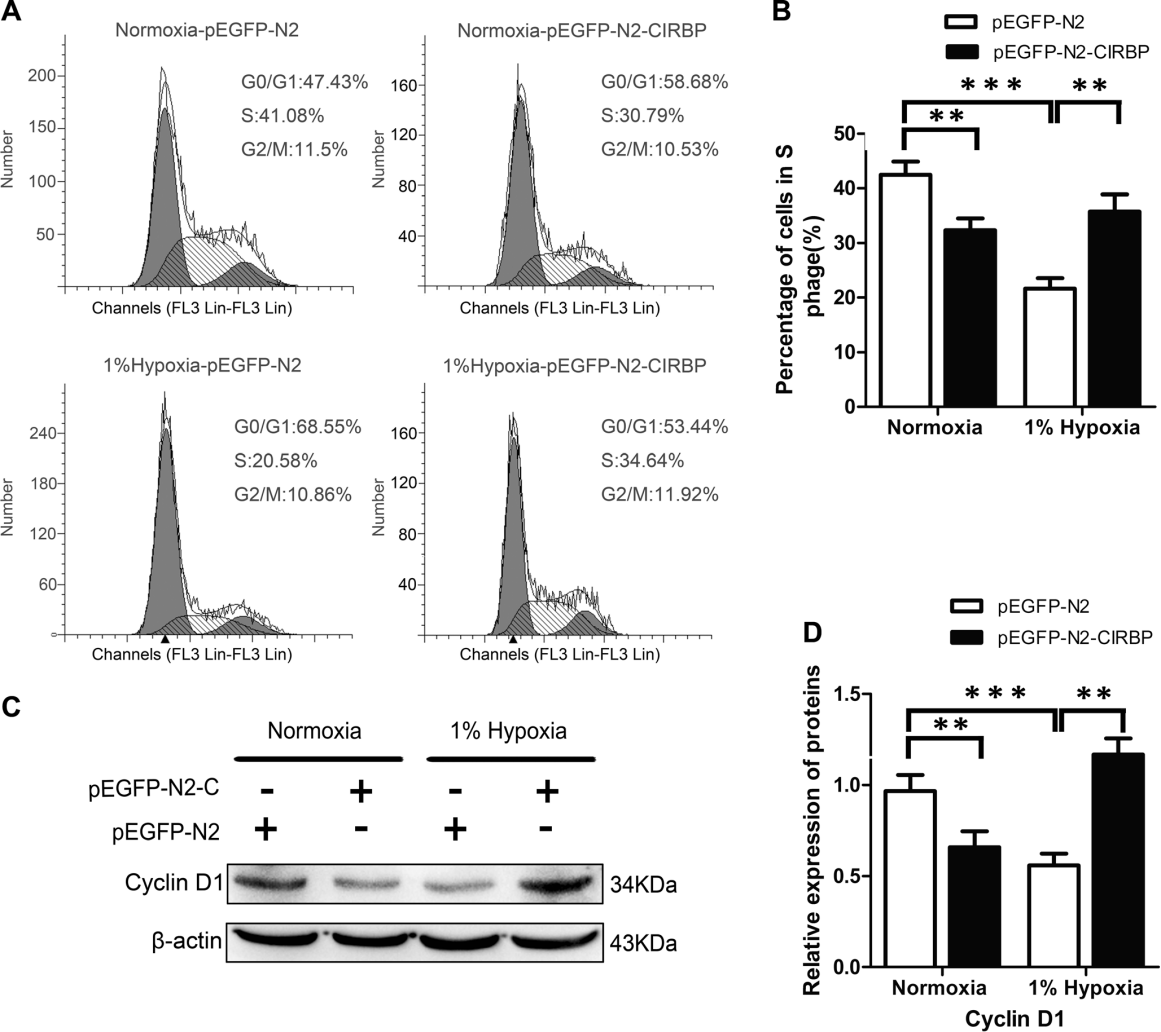
Effect of CIRBP overexpression on hypoxia-induced cell cycle arrest of C17.2 NSCs. **a**, **b** Cell cycle analysis of C17.2 NSCs transfected with mock vector (pEGFP-N2) or pEGFP-N2-CIRBP (pEGFP-N2-C) that were cultured in normoxia or 1 % O_2_ for 24 h. A representative plot of cell cycle from one of three independent experiments is shown (**a**). The first *gray* area (*close to Y axis*), the *hatched* area, and the second *gray* area (*distant to Y axis*) represent the number of cells in G1/G0, S phase, and G2 phase, respectively. Percentages of cells in G1/G0, S, and G2/M phase in control and hypoxia-exposed groups were calculated with data from three independent experiments (**b**). **c**, **d** Western blotting analysis of Cyclin D1 in transfected cells cultured in normoxia or hypoxia for 24 h. β-actin served as a protein loading control. ** and *** indicate significant difference between compared groups (**p < 0.01; ***p < 0.001)

CIRBP has been shown to stabilize Cyclin D1 and accelerate cell cycle progression from G0/G1 to S phase in cultured mouse embryonic fibroblasts [26]. To examine the mechanism underlying CIRBP restoration of cell growth of 1 % O_2_-exposed NSCs, we analyzed the cell cycle distribution and the expression of Cyclin D1 in C17.2 NSCs. Under 1 % O_2_ exposure, transfection of p-EGFP-N2-CIRBP plasmid significantly increased the percentages of cells in S phase from 21.08 to 37.43 % (Fig. 5a, b). Of note, in normal culture condition, transfection of p-EGFP-N2-CIRBP plasmid significantly reduced the percentages of cells in S phase from 40.62 to 30.70 % (Fig. 5a, b). Furthermore, the expression of Cyclin D1 was significantly enhanced by CIRBP overexpression in hypoxia-treated cells (Fig. 5c, d). In normal culture conditions, overexpression of CIRBP exhibited an inhibitory effect on the expression of Cyclin D1 (Fig. 5c, d). Overall, these data suggest that overexpression of CIRBP may prevent the detrimental effects of severe hypoxia on NSC proliferation, at least partially via regulating the expression of Cyclin D1.

## Discussion

In this study, we examined the effect of 1 % O_2_ hypoxia on the proliferation of NSCs and the possible role of CIRBP in growth arrest of NSCs under this condition. By MTT assay, EdU incorporation, and cell cycle analysis, we observed a growth-stimulating effect of 5 % O_2_ and a slight effect of 3 % O_2_ on the growth of NSCs which was consistent with previous report [35]. Interestingly, we found an obvious inhibitory effect of 1 % O_2_ on both C17.2 NSCs and primarily cultured NSCs without significantly affecting cell survival. These data suggested that 1 % O_2_ may be used to model the severe hypoxia under pathological conditions as reported [32, 35, 36]. In our study, we focused on the effect of O_2_ on the proliferation of NSCs. Then by Western blotting and real time RT-PCR, we detected a decrease of CIRBP by 1 % O_2_. In the end, we overexpressed CIRBP and found that CIRBP could restore the growth of NSCs under 1 % O_2_ culture condition. These results, for the first time, revealed a role of CIRBP in the regulation of proliferation of NSCs.

During development, NSCs live in a hypoxic microenvironment [37–39]. Mild hypoxia has been demonstrated to facilitate the proliferation of NSCs through HIF-1α and beta-catenin signaling [8, 40]. Mild hypoxia has been even adopted for improving stem cell culture [41]. Severe hypoxia, which usually occurs under pathological conditions, reportedly induces NSCs to a quiescent state or apoptosis [42]. In this study, we demonstrated that 1 % O_2_ could induce a quick growth arrest of NSCs, suggesting a detrimental effect of severe hypoxia on NSCs, and raising the importance of further investigating the mechanism of severe hypoxia on NSCs.

CIRBP is a stress responsive gene, which is expressed in almost all the tissues. In tumor cells, CIRBP is highly expressed and thought to be an oncogene [25, 27]. In cultured mouse embryonic fibroblasts, CIRBP protein can regulate the phosphorylation and stabilization of p27 and Cyclin D1 through interacting with Dyrk1b/Mirk and accelerate cell cycle progression from G0 to G1 phase as well as from G1 to S phase [26]. Our data showed that the expression of CIRBP was inhibited in the NSCs stressed by 1 % O_2_ when cell proliferation was arrested. Overexpression of CIRBP can rescue the inhibitory effect of 1 % O_2_ on the expression of Cyclin D1 and progress of cell cycle, indicating a role of CIRBP in regulating the proliferation of NSCs.

The expression of CIRBP is regulated by various stressing factors, such as hypothermia, UV irradiation, and hypoxia [15, 17, 25, 43], to meet the functional requirement of cells under different conditions, and therefore varies depending on the injury models. In conditions of hypoxia, middle cerebral artery occlusion induces the elevation of CIRBP while transient forebrain ischemia has no significant effects on the expression of CRIBP mRNA [19, 44]. In our study, the reduction of CIRBP in accompany with the arrest of cell proliferation in NSCs under severe hypoxia is consistent with the effects of hypoxia on NSC proliferation. In the hippocampus where neurogenesis persists, CIRBP decreases quickly after ischemia [19]. In vitro, H_2_O_2_ treatment can inhibit the hypothermia-induced CIRBP expression [19, 45, 46], indicating that the regulation of CIRBP by hypoxia may be reactive oxygen species (ROS)-mediated, because ROS in proper level is beneficial and ROS in higher level is toxic for NSC proliferation [45, 47, 48]. It may be possible that mild hypoxia results in mild elevation of ROS, which may then lead to CIRBP expression, while severe hypoxia induces overload of ROS, which suppresses the expression of CIRBP. Further studies are too worthy to be conducted to explore this issue.
